# Chickenpox vaccination

**DOI:** 10.1186/1824-7288-40-S1-A3

**Published:** 2014-08-11

**Authors:** Giovanni Vitali Rosati

**Affiliations:** 1 Florence, Italy

## 

Varicella is a common viral disease affecting almost the entire birth cohort. Some cases of varicella can be serious, with 2-6% of complications. The hospitalization rate for varicella in Europe ranges from 1.3 to 4.5 per 100,000 population/year and up to 10.1% of hospitalized patients report permanent or possible permanent sequelae.

In countries where routine childhood vaccination against varicella has been implemented, it has had a positive effect on disease prevention and control. Mathematical models indicate that this intervention strategy may provide economic benefits for the individual and society[1].

Despite this evidence and recommendations for varicella vaccination by official bodies such as the World Health Organization and scientific experts in the field, in Italy the last National Plan for Vaccine Prevention 2012-2014 says that vaccination against chickenpox will be included among those active and free offer for all new born Italian from 2015.

Actually only eight Italian Regions under-toke universal varicella vaccination programs, in many cases with widespread use of the vaccine MPRV.

The data of Tuscany[2], where the universal vaccination began in the end of 2008 using the quadrivalent vaccine MPRV co-administered with meningococcal C, show a marked reduction of chickenpox in the 2009-2011 period, compared to the prevaccination era (2005-2007). The avoided cases are estimated approximately 42,000 and the hospitalizations are decreased from 3.5 / 100,000 to 2,6 / 100,000. The varicella avoided hospitalizations can be evaluated as 115 in three years.

The reactogenicity data regarding the quadrivalent vaccine measles-mumps-rubella-varicella (MPRV) are discussed because an increased relative risk of febrile seizures after this vaccination, respect to the administration separate and simultaneous MMR and varicella monovalent, is reported.

The Tuscan pharmacovigilance data on MPRV in the last three years 2009-2011, did not indicate any increase of the risk of adverse events compared to the administration of the separate two preparations.

For these reasons, although a note of the Ministry of Health in 2012 invited to use vaccination separate MMR + V in the first dose, Tuscany decided to continue to use MPRV vaccine also in the first dose.
